# Supplementary material for: Short-Time Impact of Soil Amendments with Medicago Plant Materials on Soil Nematofauna
Source: Plants (Basel). 2021 Jan 12;10(1):145. doi: 10.3390/plants10010145 (PMC7828159; doi:10.3390/plants10010145)
Supplement: Supplementary file 1 [file plants-10-00145-s001.pdf]

Table S1. Total abundance (individuals 500 g^{-1} soil)[1] of nematode genera in soil amended with 10, 20 or 40 g kg^{-1} soil rates of dry biomasses from the six tested *Medicago* species.

Genera	cp rank[2]	Nontreated	Plant species																	
			M. heyniana			*M. hybrida*			*M. lupulina*			*M. murex*			*M. sativa*			*M. truncatula*		
			10	20	40	10	20	40	10	20	40	10	20	40	10	20	40	10	20	40
**Bacterivores**																				
Acrobeles	2	221	123	93	48	19	94	169	61	113	35	103	59	79	82	61	16	115	328	98
Acrobeloides	2	1345	350	419	261	83	95	847	1790	945	1010	819	1899	1355	1373	866	254	209	271	177
Alaimus	4	24	23	-	6	-	-	3	-	11	10	-	-	-	16	17	21	13	11	-
Cephalobus	2	690	66	327	414	120	135	430	46	233	302	851	598	522	562	624	714	458	103	179
Cervidellus	2	171	6	11	11	-	-	-	6	18	28	2	5	-	15	8	12	22	32	4
Diploscapter	1	113	54	24	86	63	170	28	89	150	131	36	118	35	16	41	66	113	33	52
Eucephalobus	2	15	-	18	42	10	-	-	-	12	14	15	34	222	18	-	-	-	-	-
Chiloplacus	2	114	48	49	12	6	51	53	46	38	48	69	19	57	58	36	4	353	44	46
Mesorhabditis	1	154	2022	1094	1375	1590	1163	1272	61	81	94	51	44	36	38	52	140	38	45	247
Eumonhystera	2	3	12	6	4	7	36	65	2	32	27	28	21	6	3	18	6	40	11	15
Panagrolaimus	1	36	86	58	208	113	45	28	240	217	78	99	29	14	25	22	64	853	123	158
Plectus	2	30	30	16	17	23	31	21	139	170	67	820	616	798	539	395	501	80	26	40
Rhabditis	1	120	460	245	529	253	265	315	746	860	1720	237	285	258	844	2347	788	849	701	889
Wilsonema	2	14	-	-	-	-	-	-	4	13	18	-	-	-	5	15	-	22	17	-
Zeldia	2	58	13	7	13	28	60	14	110	21	20	67	49	38	74	10	10	152	85	80
**Fungivores**																				
Aphelenchoides	2	438	50	110	63	174	118	175	1046	876	1127	156	93	74	465	953	849	297	334	389
Aphelenchus	2	383	938	1134	1397	420	866	859	958	927	1607	514	1600	728	1382	987	528	847	526	400
Ditylenchus	2	-	-	-	-	-	-	-	29	-	21	12	13	4	9	24	-	-	-	-
**Omnivores**																				
Aporcelaimellus	5	103	17	8	29	88	45	13	28	19	54	74	74	74	96	27	55	85	42	52

Dorylaimus	4	24	-	18	28	10	29	-	2	-	-	19	61	56	25	8	11	46	6	36
Ecumenicus	4	39	-	16	21	17	12	-		9	-	35	11	23	27	61	-	101	63	50
Eudorylaimus	4	27	23	10	22	88	17	97	41	34	72	83	58	119	59	32	81	11	47	12
Microdorylaimus	4	91	41	13	8	37	28	23	11	25	-	79	58	37	20	11	3	199	91	2
**Plant parasites**																				
Geocenamus	3	243	173	240	35	114	155	59	91	77	78	1392	1311	1241	182	126	98	3338	2307	2353
Meloidogyne	3	2372	304	155	145	510	384	295	91	77	38	477	365	249	132	117	92	475	590	617
Pratylenchus	3	5	4	3		3	-	-	27	11	18	23	15	75	-	-	-	-	-	-
**Root-fungal feeders**																				
Boleodorus	2	30	-	19	-	8	24	6	-	19	-	35	11	14	9	9	20	25	30	66
Filenchus	2	374	23	12	38	8	72	32	84	74	24	8	19	13	53	28	43	40	90	106
**Predators**																				
Trypila	3	14	14	4	10	13	12	21	17	23	11	40	8	52	10	10	5	54	6	8
Mylonchulus	4	37	6	10	11	7	27	-	8	16	14	9	19	3	6	-	14	20	10	17
Oxydirus	5	24	-	-	-	-	-	-	6	-	30	7	-	-	4	4	4	20	2	11

[1] Values are the sum of individuals from five 100 g^{-1} soil replicates; [2] rank value in the 1 to 5 colonizers-persisters‘ scale.
